# La dolce vita: fueling chimeric antigen receptor (CAR) T cells with Glut1 to improve therapeutic efficacy

**DOI:** 10.1097/IN9.0000000000000055

**Published:** 2025-01-13

**Authors:** Karen Slattery, David K. Finlay, Phillip K. Darcy

**Affiliations:** 1 School of Medicine, Trinity Translational Medicine Institute, St. James’s Hospital, Dublin, Ireland; 2 School of Biochemistry and Immunology, Trinity Biomedical Sciences, Trinity College Dublin, Dublin, Ireland; 3 School of Pharmacy and Pharmaceutical Sciences, Trinity Biomedical Sciences, Trinity College Dublin, Dublin, Ireland; 4 Cancer Immunology Program, Peter MacCallum Cancer Centre, Melbourne, Victoria, Australia; 5 Sir Peter MacCallum Department of Oncology, The University of Melbourne, Parkville, Australia

**Keywords:** chimeric antigen receptor T cells, metabolism, cancer, tumor, Glut1, glucose, glycolysis, cytokines

## Abstract

The approval of chimeric antigen receptor (CAR) T cell therapies for the treatment of hematological cancers has marked a new era in cancer care, with seven products being FDA approved since 2017. However, challenges remain, and while profound effects are observed initially in myeloma, the majority of patients relapse, which is concomitant with poor CAR T cell persistence. Similarly, the efficacy of CAR T cell therapy is limited in solid tumors, largely due to tumor antigen heterogeneity, immune evasion mechanisms, and poor infiltration and persistence. In this recent study, Guerrero et al endeavor to improve the efficacy of human CAR T cells by overexpressing the glucose transporter *GLUT1* and show that *GLUT1* overexpressing CAR T cells have improved capacity to persist and control tumor burden in vivo.

Metabolism plays an essential role in driving T cell anti-tumor responses ^[1]^, and so the metabolic enhancement of chimeric antigen receptor (CAR) T cells has emerged as an exciting approach to improve cell therapy ^[2]^. This study explored the mechanisms underlying the beneficial therapeutic effects of *GLUT1* overexpression (GLUT1OE) in two human CAR T cell tumor models directed against CD19 or GD2 antigens ^[3]^. In brief, GLUT1OE boosted CAR T cell glycolysis, oxidative phosphorylation (OxPhos), and glutathione metabolism, and this was associated with increased expression of cytokines such as IL17 and reduced signs of exhaustion. GLUT1OE CAR T cells were more effective at controlling tumor growth in vivo than control CAR T cells, and antigen-specific anti-tumor activity was retained 60 days post-infusion.

A striking observation from this work is that GLUT1OE had a broad impact on many aspects of cellular metabolism in CAR T cells far beyond glycolysis. Metabolomic and RNA sequencing analyses highlighted amino acid, purine, and pyrimidine metabolism as key pathways impacted by GLUT1OE, while glycolysis was not in the top 25 enriched pathways. U^13^C glucose tracing data showed that glucose was metabolized to lactate through aerobic glycolysis and to the nucleoside inosine via the pentose phosphate pathway, while a small portion was converted to isocitrate via the tricarboxylic acid (TCA) cycle (Figure 1). This suggests that GLUT1OE CAR T cells largely utilize glucose for anabolic pathways to produce biosynthetic precursors, likely to facilitate their antigen-induced proliferation, which is in agreement with what has been observed previously in CD8 T cells in in vivo ^13^C-glucose tracing experiments ^[4]^. In terms of catabolic pathways, U^13^C glucose tracing showed minimal fueling of the TCA cycle at this timepoint, possibly due to glutamine anaplerosis ^[5]^; however, mitochondrial β-oxidation was an enriched pathway in the GLUT1OE CD19-CAR T cells, which has been linked with memory T cell formation and longevity ^[1]^. The authors also provide compelling evidence that GLUT1OE promoted antioxidant defenses, including increased glutathione levels, reduced mitochondrial reactive oxygen species, and increased resistance to H_2_O_2_ exposure. Overall, these analyses highlight the profound impact that overexpression of a single nutrient transporter can have on many aspects of CAR T cell metabolism.

Arguably the most critical finding of this study is that GLUT1OE GD2-CAR T cells increased tumor control in a NALM6 leukemia model, and this is strongly supported by a similar study that over-expressed *GLUT1* in 19-28z and IL13Rα2-BBz CAR T cells ^[6]^. Administration of GLUT1OE CD19-CAR T cells modestly reduced tumor burden; however, treatment with GLUT1OE GD2-CAR T cells resulted in complete and long-lasting tumor control. GLUT1OE GD2-CAR T cells had higher rates of OxPhos than either of the CD19-CAR T cells, which may have contributed to their superior longevity in vivo. Although the efficacy of GLUT1OE CAR T cells against solid tumors was not extensively evaluated, Shi et al ^[6]^ recently showed that GLUT1OE enhanced the potency of CAR T cells in models of renal cell carcinoma and glioblastoma, suggesting that this metabolic enhancement shows promise for promoting the activity of CAR T cells against solid tumor cancers.

While this study has shed light on the specific metabolic pathways impacted by GLUT1OE in CAR T cells, it also raises important questions regarding the exact mechanism by which GLUT1OE results in improved anti-tumor capacity. The extent to which GLUT1OE increases glucose influx through GLUT1 requires further investigation, as the authors employed the non-specific glucose uptake assays 2-NBDG and deoxy-D-[1,2-3H (*N*)]-glucose, the former of which has been shown to not be taken up through GLUT1 ^[7]^. A recently developed click chemistry-based assay of 6-azido-6-deoxy-d-galactose uptake would provide more accurate data on the activity of Glut1 ^[8]^, while further quantitative analysis of glucose depletion from supernatants would reveal exactly how much additional glucose is taken up because of GLUT1OE. U^13^C glucose tracing analysis showed that glucose was indeed taken up, but whether there was some contribution by GLUT3, that had elevated expression in GLUT1OE CAR T cells, remains to be explored. As GLUT3 has a higher affinity for glucose than GLUT1, its overexpression may be even more effective than GLUT1OE, and indeed GLUT3OE in human T cells has shown therapeutic efficacy in a model of melanoma ^[9]^. It will be important in future studies to explore the therapeutic impact of GLUTOE-mediated increased cytokine production (Figure 1). The induction of T_H_2 cytokines by GLUTOE in GD2-CAR T cells is of high interest given the recent study showing that Fc−IL-4 drives CD8 T cell glycolysis and anti-tumor responses in vivo ^[10]^. Similarly, as IL17 has been shown to have both pro- and anti-tumor effects ^[11]^, the induction of T_H_17 cytokines by GLUTOE in CD19-CAR T cells requires further investigation, and defining its role in driving the therapeutic efficacy of GLUTOE CD19-CAR T cells will provide important insight into the role that IL17 plays in cancer.

This work adds to the interesting debate of whether glucose and other nutrients are truly limiting in the human tumor micro-environment (TME). While glucose competition is regularly cited as a key immunosuppressive mechanism in the TME ^[12]^, data supporting this in human tumors is scarce. In renal cell carcinoma, glucose concentrations are similar between tumor interstitial fluid (~0.5–4 mM) and normal kidney interstitial fluid (~0.5−1.5 mM) ^[13–15]^. Further, the widespread use of positron emission tomography scans to trace tumors supports the argument that glucose may not be limiting in human tumors, although this is likely to be patient and cancer dependent. GLUT1 has a low Km of ~2 mM ^[16]^, which is much lower than the concentration of glucose in the blood (5−7 mM) or in RPMI (11.1 mM). As much of the experimental work conducted in this study was in glucose-replete conditions, this suggests that GLUT1 protein expression may be the true bottleneck for glucose metabolism in CAR T cells, rather than extracellular glucose availability. If so, this raises important questions regarding what is restricting GLUT1 expression, and whether this could be targeted for therapeutic benefit. As the expression of GLUT1 at the plasma membrane is largely regulated by endosomal recycling, GLUT1 may be prevented from trafficking to the plasma membrane. Interestingly, switching the concentration of glucose from 1.5 mM to 15 mM has been shown to trigger GLUT1 endocytic trafficking to the lysosome in HeLa cells ^[17]^, suggesting that the high concentrations of glucose found in RPMI may restrict GLUT1 expression at the plasma membrane.

Overall, this is an interesting study that provides compelling evidence that enhancing the metabolic fitness of CAR T cells is a promising approach to improving their efficacy against cancer. This work and the other similar studies published this year ^[3,6,9,18,19]^ likely signal a new wave of immunometabolism research wherein the scientific discoveries made over the past decade will be harnessed to promote the advancement of cell therapies in the clinic. Given the clinical observation that leukemia patients who receive CAR T cells with better mitochondrial fitness have improved CAR T cell persistence and response to treatment ^[20]^, we anticipate that the metabolic engineering of CAR T cells may pave the way toward effective and durable cell therapies for the treatment of human cancer and unleash their potential against solid tumors.

## Figures and Tables

**Figure 1:**
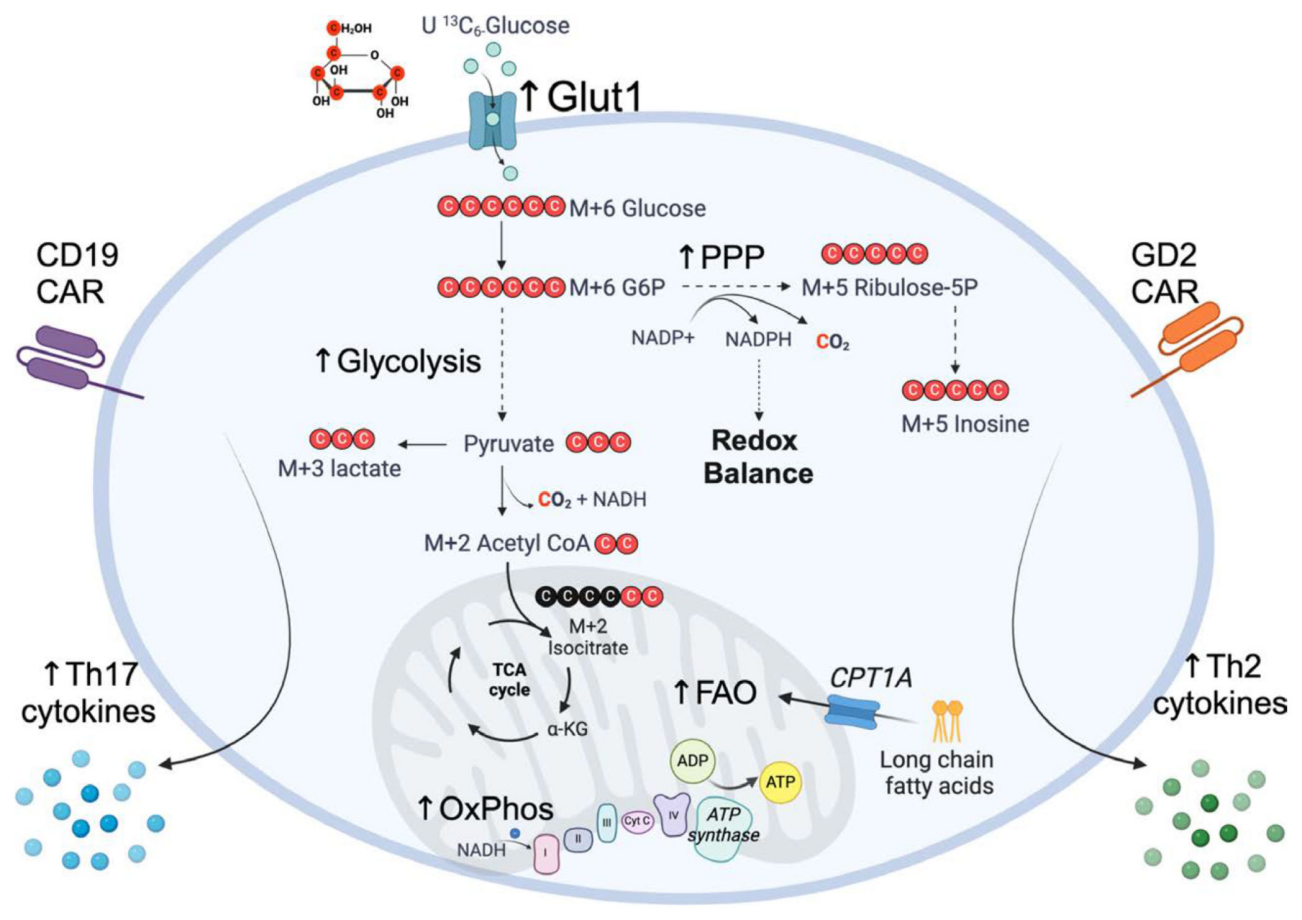
Glut1 overexpression enhances the metabolic and cytokine responses of CAR T cells. U ^13^C_6_-glucose tracing shows the fueling of glycolysis and the PPP, with minimal fueling of the TCA cycle. This leads to increased rates of glycolysis and OxPhos and production of T_H_17 cytokines in CD19 CAR T cells and T_H_2 cytokines in GD2 CAR T cells. CAR, chimeric antigen receptor; FAO, fatty acid oxidation; G6P, glucose-6-phosphate; OxPhos, oxidative phosphorylation; PPP, pentose phosphate pathway; ribulose-5-P, ribulose-5-phosphate; TCA, tricarboxylic acid. Created in https://BioRender.com.
